# Reflections on changeability *versus *stability of health-related quality of life: distinguishing between its environmental and genetic components

**DOI:** 10.1186/1477-7525-6-89

**Published:** 2008-11-02

**Authors:** Mirjam AG Sprangers, Carolyn E Schwartz

**Affiliations:** 1 Department of Medical Psychology, Academic Medical Center, University of Amsterdam, Meibergdreef 15, 1105 AZ Amsterdam, The Netherlands; 2 DeltaQuest Foundation, Inc., Concord, MA, USA; 3 Departments of Medicine and Orthopaedic Surgery, Tufts University School of Medicine, Boston, MA, USA

## Abstract

The field of health-related quality of life (HRQOL) could benefit from a broadening of perspectives to include recent advancements in research on adaptation, positive psychology, and genetics. These advances shed new light on the extent to which HRQOL is changeable or fixed. The objective of this paper is to integrate these insights and to discuss their implications for HRQOL research. We describe the Hedonic Treadmill theory, which asserts that positive events only temporarily affect happiness since people quickly return to hedonic neutrality. New empirical evidence suggests important revisions of this theory, providing a more optimistic picture of the possibility for change. Advances in positive psychology show that relatively simple interventions have the power to induce a sustainable increase in levels of happiness. Finally, a small but growing number of studies have found independent genetic influences in well-being, life satisfaction, perceived health, and even HRQOL. Given the increasing empirical evidence that HRQOL can be sustainably enhanced and is in part genetically determined, it may be useful to consider HRQOL as a concept that has state (environmental) and trait (genetic) components. This distinction will allow us to explore new pathways of improving theory, methods, and clinical practice. The overarching novel questions concern the extent to which HRQOL components are environmentally or genetically determined, and which factors lead to lasting improvement. This distinction begs for new research approaches, such as time-sampling techniques and interdisciplinary research investigating the genetic variants of HRQOL. Distinguishing between those aspects that are amenable to change from those that are relatively fixed and stable will help better target specific support interventions.

## Background

The field of quality of life outcomes in health has been developed in a relative vacuum, which can be seen as one of its major weaknesses. The field could benefit from a consideration of the convergence of knowledge and consequent insights from such diverse research areas as adaptation, positive psychology, and genetics. Although disparate, these areas of research have in common that they provide new insights into the changeability of quality of life (i.e., research on adaptation and positive psychology) versus its stability (i.e., genetic research). The objective of this paper is to highlight recent advances in these areas of research insofar as they support a state (i.e., environmental) and trait (i.e., genetic) conceptualization of quality of life and to start discussing their implications for quality-of-life research in health. As a caveat, we would like to note that the choice of these distinct research areas is not intended to be comprehensive and that we cannot and do not claim to pay credit to the depth and richness of these research fields. Our ultimate aim is to stimulate thinking and discussion about these and other implications of the state-trait conceptualization to further the field of quality-of-life outcomes in health.

Before we continue, we will first define the key components. The areas on adaptation and positive psychology are primarily targeted at happiness. Happiness has several meanings in popular discourse as well as in scholarly literature, as Diener [1] noted: "For example, happiness can mean a general positive mood, a global evaluation of life satisfaction, living a good life, or the causes that make people happy, with the interpretation depending on the context". A closely related, but distinct concept is quality of life, which usually refers to the degree to which a person's life is desirable versus undesirable. The term may include the circumstances as well as the person's perceptions, thoughts, feelings, and reactions to those circumstances [1]. In the area of health we are primarily interested in evaluating those aspects of quality of life that are affected by disease or treatment, hence the term health-related quality-of-life. Health-related quality-of-life (HRQOL) is still a loose definition and its relevant aspects may vary from study to study. However, there is consensus that it should include physical, emotional and social functioning as well as an overall evaluation of one's life quality [2]. Happiness as a global evaluation of life satisfaction and overall quality of life can therefore be seen as an important aspect of emotional well-being and thus relevant to a key dimension of HRQOL.

The three areas of research have primarily focused on the emotional component of HRQOL. Whereas the areas on adaptation and positive psychology are principally targeted at positive emotional states, genetic research is focused on both positive and negative emotional states and increasingly on subjective evaluations of physical functioning (e.g. pain and fatigue). The central theme of this paper thus extrapolates these findings to other domains and applies the state-trait conceptualization to the more inclusive concept of HRQOL.

### Adaptation theory: the Hedonic Treadmill revisited

The Hedonic Treadmill theory is a so-called "set-point" theory, which posits that individuals may have a set point for their levels of happiness. This happiness set point is assumed to be genetically determined and is likely to reflect stable and relatively immutable personality traits. These traits may influence the upper and lower limits of happiness that a given person may experience, thus establishing his or her set range. The set point can be defined as the expected value within the person's set range [3]. In their seminal 1971 chapter describing the Hedonic Treadmill model, Brickman and Campbell [4] highlighted research that emotional adaptation was similar to sensory adaptation, where novel stimuli (e.g., a nice perfume or an awful aftershave) caused perturbations initially that faded in a short time as the olfactory receptors became saturated. Similarly, people adapt to new life circumstances, and eventually return to "hedonic neutrality." This notion implies that individual and societal efforts to increase long-term levels of happiness are destined to failure.

Recent empirical evidence suggests, however, that the Hedonic Treadmill model is in need of revision. Diener et al. [5] suggested the following five important revisions. First, individuals' set points are not hedonically neutral. In fact, most people are happy most of the time, implying that their emotional set point is a positive and not a neutral one. Second, people have different set points that are partly determined by their personality. Personality factors may thus facilitate or inhibit higher levels of well being. Third, a single person may have multiple happiness set points (e.g., for family, marital, or work life). Changes in one domain do not necessarily coincide with changes in another domain, and positive and negative emotional responses to different domains can co-exist [6]. Fourth, individuals differ in the rate and extent of adaptation to similar events, with some individuals changing their long-term levels of happiness and others not at all. Finally, individuals can learn skills to sustainably enhance their levels of well-being [7,8].

Thus, individual differences in personality style, emotional responsiveness, and skills mediate and mitigate the role that adaptation can play on happiness. These identifiable and measurable factors can determine the extent to which changes are sustainable. Diener and colleagues' revisions of the Hedonic Treadmill model create a more flexible and nuanced representation of what is changeable and what is fixed between and within people.

### Refocusing may change chronic levels of happiness

Work by Kahneman and colleagues [9] provide an opening explanation for change in an individual's chronic level of happiness. They note that a specific life circumstance only influences a person's well being if it draws that person's attention. If it is novel [10] or unexplainable [11], then people pay attention. Their attention fades when they get used to the circumstance or have found a satisfactory explanation for it. Sometimes people engage in a "focusing illusion", where they focus on a single factor thereby exaggerating its influence or importance [9]. This focusing can be the result of questionnaire framing, such as the order of questions (e.g., "How severe is your pain?", "How satisfied are you with your quality of life?") [12-14] or framing life circumstances due to a recent salient change (e.g., a car accident involving nerve damage). The focusing illusion helps to explain those counter-intuitive results of HRQOL research that disabled people are happier than you think, such that the healthy observer focuses on the disability whereas the disabled person is taking all aspects of his/her life into account and not merely his/her disability. We will see below that if one were to help people to refocus on the positive aspects of life, then one may be able to raise their chronic level of happiness.

### Positive psychology: sustainable change in long-term happiness

Lyubomirsky's recent intervention work can been seen as an example of such re-focusing. Her conceptual model of happiness [3] proposes that one's chronic happiness level is determined by three classes of factors: (1) a genetically-determined set point for happiness; (2) circumstantial factors, such as living in a safe environment; and (3) intentional activities. Whereas the genetic class is assumed to be stable over time, circumstantial factors are susceptible to change but exert only a small effect. In contrast, intentional activities are modifiable behavioral, cognitive, or volitional activities that can have a measurable and sustainable impact on subjective happiness [15].

The success of this latter mutable class of factors relies on the individuals' level of commitment and consistency, and the fundamental match with their values and goals. Recent empirical work has documented that interventions based on simple and popular concepts such as committing acts of kindness [16], visualizing one's best possible future selves [17], expressing gratitude [17-19] or forgiveness [19], and thoughtful self-reflection [15] had the power to induce a sustainable increase in levels of happiness. Although most work to date has been based on intervention studies of healthy psychology undergraduates, other researchers have documented sustainable increases in quality of life or well-being using some of these very same intervention concepts in samples varying in age, health status, and risk factors [20]. For example, recent research on altruism has documented physical and  mental health benefits and reduced mortality in people who regularly engage  in acts of kindness [21-23].

### Genetic research: genetic predisposition of well-being

#### Heritability Research on Twins

Research using a classical twin design (comparing identical and fraternal twins reared together and/or apart) and structural equation modelling has provided empirical evidence of a genetic predisposition for psychological attributes related to quality of life, including negative (e.g. depression, anxiety, psychosocial distress) and positive (e.g. well-being, life satisfaction, happiness) emotional states. For example, the classic Minnesota study of separated twins indicated that 40–55% of the variability in negative and positive emotionality is due to genetic variance [24]. To illustrate further, Rijsdijk and colleagues [25] found that in a sample of 1950 UK female twin pairs, 20% to 44% of the level of psychological distress was heritable. Additionally, there is increasing evidence that genetic factors account for substantial individual variations in subjective well-being [26-30] and life satisfaction [31,32].

Since personality traits are biologically-based, enduring dispositions that are 30% to 50% heritable [33], they are of particular interest for studying the genetic underpinning of subjective well-being. A large body of literature indicates that personality traits are substantially related to subjective well-being: the variance of subjective well-being explained by personality can reach as high as 39% to 63% [34]. Weiss and colleagues [35] moved this research line a step further by examining whether subjective well-being and personality share the same genetic structure. Using a representative sample of 973 twin pairs, they demonstrated that the genetic variance underlying individual differences in subjective well-being also accounted for individual differences in personality. In other words, there were no genetic effects unique to subjective well-being; they were all shared with personality.

Genetic influences have also been reported for self-rated health [30,36-44]. Typically in these studies, health is assessed with a single item, e.g., "What is your health like, at present?" [30]. To our knowledge, only one study examined the heritability of health as measured by a more comprehensive and responsive tool, the SF-36 [39]. This study used data from a normally distributed community cohort of 2928 male twin members. Results indicated moderate genetic effects (17 to 33% of the variance) on the eight SF-36 domains and the physical and mental health summary measures.

#### Genetic Research

Genetic research has not yet identified the genes that are involved in quality of life or self-rated health. Genetic research, however, has been successful in identifying chromosomal regions and genetic variants for related attributes, such as depression [45], cognition [46], and pain [47]. For example, there is consistent and increasing evidence that DNA sequence variations in the region of chromosome 15q influences susceptibility for unipolar depression [45,48-59]. To illustrate further, the epsilon4 allele of the APOE gene has been investigated for association with health-related outcomes and has been found to be related to normal cognitive aging [46]. Finally, catechol-O-methyltransferase has been found to be a key regulator of affective mood, cognitive function, and pain perception [47,51,52]. Other translational research has explored the genetic basis for physical response to treatment and patient survival. But the degree to which genetic structure impacts psychosocial response to a diagnosis is still unknown.

To date, only Sloan and colleagues [53] have examined the direct link between polymorphisms and HRQOL in cancer patients. Using data of 494 patients enrolled in a randomized North Central Cancer Treatment Group (NCCTG) clinical trial, they found preliminary evidence for relationships between overall quality of life, symptom distress, and fatigue with variant genotypes of two enzymes involved in folate metabolisms. They took a skeptical approach to the analysis and pre-specified a clinically meaningful effect size that would have to be observed to indicate a potential relationship. HRQOL scores between patients with normal and variant genotypes were compared, controlling for potential confounding variables of age, sex, performance status, and previous adjuvant chemotherapy. More than triple the number of relationships between the measured polymorphisms and HRQOL outcomes were observed than would be expected by chance alone. Clearly, such promising preliminary results need further validation with large-scale studies.

The heritability studies on twins and the emerging genetic studies are sufficiently compelling to justify the assumption that there is a genetic component to the different domains of HRQOL.

### Implications for HRQOL research

The pursuit of happiness is an age-old quest, and the means for its attainment differ by discipline, historical period, and values of the individual. The empirically-based revision of the treadmill model highlights that the personal level of happiness is more flexible and thus changeable than was previously thought. There is mounting evidence that sustainable increases in happiness levels are possible via interventions that teach ways of refocusing one's perspective and priorities, and that these increases are sustained over time. Although this newfound flexibility is heartening, it is important to recognize the genetic or predetermined constraints that limit the extent to which HRQOL can be enhanced. The convergence of these three lines of investigation thus supports a conceptualization of HRQOL as a state- and trait- induced entity. The distinction between what aspects of HRQOL are flexible or are fixed has important implications for theory, methods, and clinical practice.

#### Implications for Theory

The primary distinction of state and trait has an overarching impact on the nomological network of HRQOL. The construct of HRQOL is now known to be composed of domains that have both changeable and fixed components. Codifying the proportion of a given domain that is changeable will improve both theoretical models and measurement. For example, the state-trait ratio may vary per domain, with some domains perhaps being more flexible (psychological functioning?) than others (symptom experiences?). Moreover, when we are interested in the effects of disease, treatment, or psychological interventions on patients' HRQOL, we need to determine upfront which aspects of HRQOL can be influenced by these stimuli and which aspects not. In other words, we need to ensure that the influenceable  state components are addressed. The distinction of Fayers and Hand [54,55] between 'effect' and 'causal' indicators of HRQOL is also relevant to this discussion because previously identified effect indicators may in truth be causal indicators if the components of HRQOL (e.g. physical functioning, psychological functioning) are known to have a strong genetic component.

The field of HRQOL has never focused on that which is innate. Thus, there is a compelling need to reveal the unknown variables that play a role in HRQOL. While it is exciting that genetic research will be initiated to start understanding the genetic underpinnings of HRQOL, it is also important to note that genetic research is challenged by weak gene-disease associations [56] and inconsistency of results [57]. Finding the optimal path to uncover the relationships between genetic variants and HRQOL variables will therefore be a challenge in itself.

#### Implications for methods

The insight that HRQOL is determined by changeable and fixed factors underlines the need for more nuanced measurements and other research paradigms. For example, in intervention studies, we should target more the state rather than the trait components of HRQOL. Whereas most established HRQOL measures yield adequate levels of responsiveness, we feel this distinction merits attention. For example, state components are better captured by affective aspects of HRQOL (e.g., how good do you feel?) than by cognitive aspects (e.g., how satisfied are you?) [58,59], and are only revealed by a short time frame (e.g. the current day). A particularly interesting method is the experience-sampling technique that studies patients' HRQOL in their natural environment. Participants in such a study interrupt their ongoing activities and provide a report of their HRQOL of that moment. Patients can complete their self-reports after designated intervals, after pre-designated events, or when prompted by a randomly timed signal [60]. Since such methods are expensive, Kahneman and colleagues [61] suggested a hybrid approach, the Day Reconstruction Method, which combines a time-use study with a technique for retrieving affective experiences. Patients are asked to construct a diary of the previous day that consists of the sequence of episodes and then to describe the feelings they experienced during each episode. By definition, such methods capture the mood of a particular moment, and are thus focused on states. Rather than capturing a belief-based generic judgment as assessed by HRQOL questionnaires (e.g. "my quality of life is very good despite the chemotherapy") they tap specific episodic reports (e.g. "but yesterday I felt lousy") [61]. Such methods can also capture the diurnal rhythm of symptoms, such as fatigue, which may provide relevant information about specific treatment effects. We advocate the use of such time sampling techniques in HRQOL research to supplement common survey methods. The combination of these methods is expected to yield more useful and valid results that will also reveal the different causes of HRQOL (e.g., features of the current situation, personality characteristics, and life circumstances).

Additionally, studies are needed that directly relate gene expressions to HRQOL. This path, however, is complex considering the potential number of genes, gene interactions, and quality-of-life variables that may be involved. To date, genetic research has burgeoned thanks to technical advancements, such as high-throughput genotyping. However, in pursuing the delineation of the relationship between genes and quality of life, both genetic and quality-of-life research is hindered by a mono-disciplinary approach. Few genetic researchers are working with quality-of-life endpoints, and similarly few quality-of-life researchers are engaged in genetic research. It is therefore of paramount importance to join forces among the disparate disciplines. Not only do we need such genetic studies to help identify and quantify the role that genes play in the experience of physical and emotional well-being, but also to clarify how genes play a role in individual differences in response to medical and psychological interventions. A methodological implication of such characterizations at the genetic level might be that current HRQOL measures are not sufficiently precise for this purpose (i.e., "phenotyping" HRQOL). Increased understanding of genetic determinism will also have clear effects on the design of randomized controlled trials. Trait, and in the future genetic, information may, for example, be included as an eligibility criterion or as a stratification variable prior to randomization.

We need more intervention studies to hone methods for raising long-term levels of happiness, and to investigate whether these intention-based and altruistic interventions can influence HRQOL. Such studies should include substantial longitudinal follow-up to determine whether the sustainable increases last over many years and possibly even over developmental milestones. To date, measurable and sustainable increases in HRQOL have been achieved via positive psychology interventions, primarily in psychology undergraduates. Future research should thus design interventions that are applicable and feasible in medically ill patient populations.

#### Implications for clinical practice

We need to improve happiness levels in our patients as these can not only enhance levels of psychological well being, but can even influence health [62,63] and success across multiple life domains [64]. Insight into the environmental versus genetic components of HRQOL will allow us to explore new pathways for improving patient care. Clearly, different causes of HRQOL require different support interventions. Additionally, we may be able to identify patients who are susceptible to poor quality of life and thus better target specific support and clinical management interventions. The genetic information can be used to tailor individualized treatments for quality of life in the same manner as individualized treatments for the underlying disease itself [53]. Doctors will eventually use genetic patterns for several tasks – to tell whether, for example, a cancer will spread, to predict how various therapies such as specific drugs or radiation will work, and perhaps even to see how someone's quality of life will be affected [53]. An important clinical implication is that there are genetically determined limitations on the amount of improvement in perceived quality of life that can be influenced by treatment.

### Epilogue

Notable and novel research from three diverse fields has implications for HRQOL research. As we reflect on the meaning of state and trait facets of quality of life, it seems that our field must continue in partnership with these other areas of research, considering for example how a positive psychological intervention might work in synergy with medical intervention, and conversely, how genetics informs or constrains the possible impact on HRQOL of a medical intervention. It is our hope that these reflections will stimulate thinking and discussion to further the field of HRQOL.

## List of abbreviations used

HRQOL: health-related quality of life

## Competing interests

The authors declare that they have no competing interests.

## Authors' contributions

MAGS conceived of the review and its implications for a state-trait conceptualization of HRQOL and co-drafted the manuscript. CES helped focusing the review and its implications for HRQOL research and co-drafted the manuscript. Both authors read and approved the final manuscript.

## References

[B1] Diener E (2006). Guidelines for national indicators of subjective well-being and ill-being. Journal of Happiness Studies.

[B2] Fayers PM, Machin D (2007). Quality of Life The assessment, analysis and interpretation of patient-reported outcomes.

[B3] Lyubomirsky S, Sheldon KM, Schkade DA (2005). Pursuing happiness: the architecture of sustainable change. Review of General Psychology.

[B4] Brickman P, Campbell DT, Appley MH (1971). Hedonic relativism and planning the good society. Adaptation Level Theory: A Symposium.

[B5] Diener E, Lucas RE, Scollon CN (2006). Beyond the hedonic treadmill. Revising the adaptation theory of well being. American Psychologist.

[B6] Folkman S (1997). Positive psychological states and coping with severe stress. Social Science & Medicine.

[B7] Lyubomirsky L, Sousa L, Dickerhoof R (2006). The cost and benefits of writing, talking, and thinking about life's triumphs and defeats. Journal of Personality and Social Psychology.

[B8] Seligman MEP, Steen TA, Park N, Peterson C (2005). Positive psychology progress: empirical validation of interventions. American Psychologist.

[B9] Kahneman D, Krueger AB, Schkade DA, Schwarz N, Stone AA (2006). Would you be happier if you were richer? A focusing illusion. Science.

[B10] Kahneman D, Thaler RH (2006). Anomalies: attention and experienced utilities. Journal Economic Perspective.

[B11] Wilson TD, Gilbert DT (2005). Affective forecasting. Knowing what to want. Current Directions in Psychological Science.

[B12] Kahneman D, Tversky A (1982). The psychology of preferences. Scientific American.

[B13] Kahneman D, Tversky A (1983). Choices, values, and frames. American Psychologist.

[B14] Llewellyn-Thomas HA, McGreal MJ, Thiel EC (1995). Cancer patients' decision making and trial entry preferences: the effects of "framing" information about short-term toxicity and long-term survival. Medical Decision Making.

[B15] Sheldon KM, Lyubomirsky S (2006). Achieving sustainable gains in happiness: Change your actions, not your circumstances. Journal of Happiness Studies.

[B16] Otake K, Shimai S, Tanaka-Matsumi J, Otsui K, Fredrickson BL (2006). Happy people become happier through kindness: A counting kindnesses intervention. Journal of Happiness Studies.

[B17] Sheldon KM, Lyubomirsky S (2006). How to increase and sustain positive emotion: the effects of expressing gratitude and vizualising best possible selves. Journal of Positive Psychology.

[B18] Emmons RA, McCullough ME (2003). Counting blessings versus burdens: an experimental investigation of gratitude and subjective well-being in daily life. Journal of Personality and Social Psychology.

[B19] McCullough ME, Pargament KI, Thoresen CE (2000). Forgiveness: Theory, Research and Practice.

[B20] Post SG (2007). Altruism & Health: Perspectives from Empirical Research.

[B21] Brown SL, Nesse RM, Vinokur AD, Smith DM (2003). Providing social support may be more beneficial than receiving it: Results from a prospective study of mortality. Psychological Science.

[B22] Schwartz CE, Meisenhelder JB, Ma A, Reed G (2003). Altruistic social interest behaviors are associated with better mental health. Psychosomatic Medicine.

[B23] Vaillant GE (2000). Adaptive mental mechanisms: Their role in a positive psychology. American Psychologist.

[B24] Tellegen R, Lykken DT, Bouchard TJ, Wilcox KJ, Segal NL, Rich S (1988). Personality similarity in twins reared apart and together. J Pers Soc Psychol.

[B25] Rijsdijk FV, Snieder H, Ormel J, Sham P, Goldberg DP, Spector TD (2003). Genetic and enviromental influences on psychological distress in the population: General Health Questionnaire analysis in UK twins. Psychological Medicine.

[B26] Bergeman CS, Plomin R, Pedersen NL, McClearn GE (1991). Genetic mediation of the relationship between social support and psychological well being. Psychology and Aging.

[B27] Lykken D, Tellegen A (1996). Happiness is a stochastic phenomenon. Psychological Science.

[B28] Newman DL, Tellegen A, Bouchard TJ (1998). Individual differences in adult ego development: sources of influence in twins reared apart. Journal of Personality and Social Psychology.

[B29] Roysamb E, Harris J, Magnus P, Vitterso J, Tambs K (2002). Subjective well being. Sex specific effects of genetic and environmental factors. Personality and Individual Differences.

[B30] Roysamb E, Neale MC, Tambs K, Reichborn-Kjennerud T, Harris JR (2003). Happiness and health: environmental and genetic contributions to the relationship between subjective well being, perceived health and somatic illness. Journal of Personality and Social Psychology.

[B31] Stubbe JH, Posthuma D, Boomsma DI, de Geus EJC (2005). Heritability of life satisfaction in adults: a twin study. Psychological Medicine.

[B32] Harris JR, Pedersen NL, Stacey C, McClearn GE, Nesselroade JR (1992). Age differences in the etiology of the relationship between life satisfaction and self-rated health. Journal of Aging and Health.

[B33] Plomin R, DeFries JC, McClearn GE, McGuffin P (2005). Behavioral Genetics.

[B34] Steel P, Schmidt J, Shultz J (2008). Refining the relationship between personality and subjective well-being. Psychological  Bulletin.

[B35] Weiss A, Bates TC, Luciano M (2008). Happiness is a personal(ity) thing: the genetics of personality and well-being in a representative sample. Psychological Science.

[B36] Harris JR, Pedersen NL, McClearn GE, Plomin R, Nesselroade JR (1992). Age differences in genetic and environmental influences for health from the Swedish adoption/twin study of aging. Journal of Gerontology.

[B37] Kendler KS, Myers JM, Neale MC (2000). A multidimensional twin study of mental health in women. American Journal of Psychology.

[B38] Romeis JC, Scherrer JF, Xian H, Eisen SA, Bucholz K, Heath AC, Goldberg J, Lyons MJ, Henderson WG, True WR (2000). Heritability of self-reported health status. Health Services Research.

[B39] Romeis JC, Heath AC, Xian H, Eisen SA, Scherrer JF, Pedersen NL, Fu Q, Bucholz KK, Goldberg J, Lyons MJ, Waterman B, Tsuang MT, True WR (2005). Heritability of SF-36 among middle-age, middle-class, male-twins. Medical Care.

[B40] Svedberg P, Lichtenstein P, Pedersen NL (2001). Age and sex differences in genetic and environmental factors for self-related health. Journal of Gerontology.

[B41] Svedberg P, Gatz M, Lichtenstein P, Sandin S, Pedersen NL (2005). Self-related health in a longitudinal perspective: a 9 year follow-up twin study. Journal of Gerontology.

[B42] Svedberg P, Bardage C, Sandin S, Pedersen NL (2006). A prospective study of health, life style and psychosocial predictors of self-rated health. European Journal of Epidemiology.

[B43] Christensen K, Holm NV, McGue M, Corder L, Vaupel JW (1999). A Danish population based twin study on general health in the elderly. Journal of Aging and Health.

[B44] Leinonen R, Kaprio J, Jylha M, Tolvanen A, Koskenvuo M, Heikkinen E, Rantanen T (2005). Genetic influences underlying self-rated health in older female twins. Journal of American Geriatric Society.

[B45] Levinson DF (2006). The genetics of depression. Biologic Psychiatry.

[B46] Deary IJ, Whiteman MC, Pattie A, Starr JM, Hayward C, Wright AF, Carothers A, Whalley LJ (2002). Cognitive change and the APOE epsilon 4 allele. Nature.

[B47] Nackley AG, Shabalina SA, Tchivileva IE, Satterfield K, Korchynskyi O, Makarov SS, Maixner W, Diatchenko L (2006). Human catechol-o-methyltransferase haplotypes modulate protein expression by altering mRNA secondary structure. Science.

[B48] Levinson DF, Evgrafov OV, Knowles JA, Potash JB, Weissman MM, Scheftner WA, DePaulo JR, Crowe RR, Murphy-Eberenz K, Marta DH, McInnis MG, Adams P, Gladis M, Miller EB, Thoma J, Holmans P (2007). Genetics of reccurent early-onset major depression (GenRED): significant linkage on chromosome 15q25-q26 after fine mapping with single nucleotide polymorphism markers. American Journal of Psychiatry.

[B49] Holmans P, Weissman MM, Zubenko GS, Scheftner WA, Crowe RR, DePaulo JR, Knowles JA, Zubenko WN, Murphy-Eberenz K, Marta DH, Boutelle S, McInnis MG, Adams P, Gladis M, Steele J, Miller EB, Potash JB, MacKinnon DF, Levinson DF (2007). Genetics of recurrent early-onset major depression (GenRED): Final genome scan report. American Journal of Psychiatry.

[B50] McGuffin P, Cohen S, Knight J (2007). Homing in depression genes. American Journal of Psychiatry.

[B51] Zubieta JK, Heitzeg MM, Smith YR, Bueller JA, Xu K, Xu Y, Koeppe RA, Stohler CS, Goldman D (2003). COMT val158met genotype affects mu-opioid neurotransmitter responses to a pain stressor. Science.

[B52] Diatchenko L, Slade GD, Nackley AG, Bhalang K, Sigurdsson A, Belfer I, Goldman D, Xu K, Shabalina SA, Shagin D, Max MB, Makarov SS, Maixner W (2005). Genetic basis for individual variations in pain perception and the development of a chronic pain condition. Human Molecular Genetics.

[B53] Sloan J, Xinghua Z (2006). Genetics and quality of life. Current Problems in Cancer.

[B54] Fayers PM, Hand D (1997). Factor analysis, causal indicators and quality of life. Quality of Life Research.

[B55] Fayers PM, Hand DJ, Bjordal K, Groenvold M (1997). Causal indicators in quality of life research. Quality of Life Research.

[B56] Khoury MJ, Little J, Gwinn M, Ioannidis JPA (2007). On the synthesis and interpretation of consistent but weak gene-disease associations in the era of genome-wide association studies. International Journal of Epidemiology.

[B57] Ioannidis JPA (2007). Non-replication and inconsistency in the genome wide association setting. Human Heredity.

[B58] de Haes JCJM, de Ruiter JH, Tempelaar R, Pennink BJW (1992). The distinction between affect and cognition in the quality of life of cancer patients – sensitivity and stability. Quality of Life Research.

[B59] Veenhoven R (2006). How do we assess how happy we are? Tenants, implications and tenability of three theories. Paper presented at conference on New Directions and International Perspectives in the Study of Happiness, University of Notre Dame, USA.

[B60] Scollon CN, Kim-Prieto C, Diener E (2003). Experience sampling: promises and pitfalls, strengths and weaknesses. Journal of Happiness Studies.

[B61] Kahneman D, Krueger AB, Schkade DA, Schwarz N, Stone AA (2004). A survey method for characterizing daily life experience: the day reconstruction method. Science.

[B62] Pressman SD, Cohen S (2005). Does positive affect influence health?. Psychological Bulletin.

[B63] Howel RT, Kern ML, Lyubomirsky S (2007). Health benefits: Meta-analytically determining the impact of well-being on objective health outcomes. Health Psychology Review.

[B64] Lyubomirsky S, King L, Diener E (2005). The benefits of frequent positive affect: Does happiness lead to success?. Psychological Bulletin.

